# *In vivo* Differential Brain Clearance and Catabolism of Monomeric and Oligomeric Alzheimer's Aβ protein

**DOI:** 10.3389/fnagi.2016.00223

**Published:** 2016-09-27

**Authors:** Farron L. McIntee, Patrizia Giannoni, Steven Blais, George Sommer, Thomas A. Neubert, Agueda Rostagno, Jorge Ghiso

**Affiliations:** ^1^Department of Pathology, New York University School of MedicineNew York, NY, USA; ^2^Department of Biochemistry and Molecular Pharmacology, New York University School of MedicineNew York, NY, USA; ^3^Kimmel Center for Biology and Medicine at the Skirball Institute, New York University School of MedicineNew York, NY, USA; ^4^Radiation Safety Office, New York University School of MedicineNew York, NY, USA; ^5^Department of Psychiatry, New York University School of MedicineNew York, NY, USA

**Keywords:** Aβ brain efflux, Aβ brain homeostasis, local proteolytic degradation, stereotaxic intra-cerebral injection, targeted mass spectrometric analysis, cerebrospinal fluid

## Abstract

Amyloid β (Aβ) is the major constituent of the brain deposits found in parenchymal plaques and cerebral blood vessels of patients with Alzheimer's disease (AD). Several lines of investigation support the notion that synaptic pathology, one of the strongest correlates to cognitive impairment, is related to the progressive accumulation of neurotoxic Aβ oligomers. Since the process of oligomerization/fibrillization is concentration-dependent, it is highly reliant on the homeostatic mechanisms that regulate the steady state levels of Aβ influencing the delicate balance between rate of synthesis, dynamics of aggregation, and clearance kinetics. Emerging new data suggest that reduced Aβ clearance, particularly in the aging brain, plays a critical role in the process of amyloid formation and AD pathogenesis. Using well-defined monomeric and low molecular mass oligomeric Aβ1-40 species stereotaxically injected into the brain of C57BL/6 wild-type mice in combination with biochemical and mass spectrometric analyses in CSF, our data clearly demonstrate that Aβ physiologic removal is extremely fast and involves local proteolytic degradation leading to the generation of heterogeneous C-terminally cleaved proteolytic products, while providing clear indication of the detrimental role of oligomerization for brain Aβ efflux. Immunofluorescence confocal microscopy studies provide insight into the cellular pathways involved in the brain removal and cellular uptake of Aβ. The findings indicate that clearance from brain interstitial fluid follows local and systemic paths and that in addition to the blood-brain barrier, local enzymatic degradation and the bulk flow transport through the choroid plexus into the CSF play significant roles. Our studies highlight the diverse factors influencing brain clearance and the participation of various routes of elimination opening up new research opportunities for the understanding of altered mechanisms triggering AD pathology and for the potential design of combined therapeutic strategies.

## Introduction

The most frequent form of amyloidosis in humans is related to the deposition of amyloid-β (Aβ) in Alzheimer's disease (AD), with cumulative biochemical, genetic, and *in vivo* data strongly suggesting a central role for this molecule in the pathogenesis of the disorder. Aβ is the major constituent of the tissue deposits found in parenchymal plaques and cerebral blood vessels of patients with AD and with Down's syndrome, the latter exhibiting a trisomy in chromosome 21 which codes for the Amyloid Precursor Protein (APP) and lead to AD pathology by middle age (Masters et al., 1985a; Busciglio et al., 2002). Indeed, Aβ is an internal processing product of this transmembrane APP precursor molecule (Querfurth and LaFerla, 2010; Rostagno et al., 2010) generated through proteolytic cleavage by the β- and γ-secretases (Masters et al., 1985b; Kang et al., 1987; Ghiso and Frangione, 2002). Although it is unclear what primarily triggers and drives the progression of AD, histopathologic, genetic, biochemical, and physicochemical studies, together with information obtained from transgenic animal models, strongly support the notion that abnormal aggregation/fibrillization, and subsequent Aβ tissue accumulation are key players in the disease pathogenesis (Mattson, 2004; Walsh and Selkoe, 2007; Querfurth and LaFerla, 2010; Holtzman et al., 2011). Although the abundance of mature amyloid plaques correlates poorly with AD severity (Lue et al., 1999; McLean et al., 1999), current data indicate that the transition from soluble monomeric species normally found in circulation to oligomeric, protofibrillar, and end-point fibrillar assemblies contribute significantly to disease pathogenesis. Intermediate oligomeric and protofibrillar forms, in particular, seem to display the most potent effects in neuronal cells, inducing synaptic disruption and neurotoxicity (Caughey and Lansbury, 2003; Walsh and Selkoe, 2007). Numerous studies have shown that these soluble oligomeric forms of Aβ—which have been identified *in vivo* and isolated from brain, plasma, and CSF (Kuo et al., 1996; Roher et al., 1996, 2000)—are capable of affecting synaptic function by various mechanisms (Galvan and Hart, 2016), impairing glutamatergic synaptic transmission strength and plasticity, altering synaptic structure (Whalen et al., 2005), reducing efficacy of synapses and causing synaptic loss (Walsh et al., 2002; Haass and Selkoe, 2007; Nicholls et al., 2008).

The process of oligomerization/fibrillization is concentration-dependent, and therefore it is highly reliant on the homeostatic mechanisms that regulate the steady state levels of Aβ modulating the delicate balance between rate of synthesis, dynamics of aggregation, and rate of brain efflux. For the majority of AD cases, which are of late-onset and of sporadic origin, the cause of this imbalance is unclear and remains a subject of active investigation. While to date no evidence supports an increase in the overall production in sporadic cases, current research suggests that an impaired clearance in late onset AD plays a critical role in the process of amyloid formation and the pathogenesis of the disease (Mawuenyega et al., 2010). Many pathways are currently being investigated, among them perivascular drainage, receptor-mediated cell uptake, blood brain barrier (BBB) transport, and local proteolytic degradation, all undoubtedly contributors to brain Aβ clearance (Deane et al., 2004, 2009; Bakker et al., 2016; Morris et al., 2016) in conjunction with the bulk flow of ISF into the CSF through the choroid plexus epithelium, which remarkably shares many of the receptors involved in BBB clearance (Dietrich et al., 2008; Behl et al., 2009) as well as the recently described paths for CSF recycling through the ISF (Iliff et al., 2012). Notably, in spite of the relevance of Aβ oligomerization for the disease pathogenesis, the vast majority of the reported Aβ clearance data have been generated with monomeric Aβ species or with peptides with poorly characterized aggregation state (Zlokovic et al., 1993; Ghersi-Egea et al., 1996; Martel et al., 1997; Mackic et al., 1998; Poduslo et al., 1999; Shibata et al., 2000; Strazielle et al., 2000; Bading et al., 2002; Devi and Ohno, 2012). The present work was designed to address this gap in knowledge and provide quantitative evaluation of the differential brain removal efficiency of pathogenic oligomeric Aβ assemblies while providing information into the relevance of the *in situ* catabolic break-down of the peptide. Based on data from intra-cerebral stereotaxic injections of well-defined monomeric and low molecular mass oligomeric Aβ assemblies in C57BL/6 wild-type mice in combination with biochemical and mass spectrometry analyses in CSF, the current work highlights the fast physiologic degradation and brain elimination of the peptide and provides a clear indication of the detrimental role of oligomerization for brain Aβ efflux. Immunofluorescence confocal microscopy studies bring insight into the cellular pathways involved in the brain clearance and cellular uptake of Aβ.

## Materials and methods

### Peptide synthesis, solubilization, and oligomerization

Aβ1–40 (DAEFRHDSGYEVHHQKLVFFAEDVGSNKGAIIGLMVGGVV) homologous to residues 672–711 of human Aβ precursor protein APP770 was synthesized by J. Elliot at the W. M. Keck Facility at Yale University using *N-tert*-butyloxycarbonyl chemistry and purified by reverse phase-high performance liquid chromatography on a Vydac C4 column (Western Analytical, Murrieta, CA). The molecular mass of the synthetic homolog was corroborated by matrix-assisted laser desorption ionization time-of-flight (MALDI-ToF) mass spectrometry, and its concentration assessed by amino acid analysis, as we previously reported (Ghiso et al., 2004). The peptide eluted as a single component from the HPLC column and exhibited an experimental molecular mass of 4329.1 Da (theoretical mass: 4329.9 Da).

Aβ1-40 was incubated at a concentration of 1 mg/ml in 1,1,1,3,3,3, hexafluoro-isopropanol (HFIP; Sigma Chemical Co., St. Louis, MO) for 24 h, a pretreatment that breaks down β -sheet structures and disrupts hydrophobic forces leading to monodisperse amyloid subunit preparations (Stine et al., 2003). The HFIP-pretreated peptide was lyophilized, subsequently reconstituted to 1 mM in deionized water followed by further dilution in 2X phosphate-buffered saline (PBS) to a final concentration of 1 μg/μl in PBS and either immediately used for radioiodination and direct intracerebral injection or maintained frozen at −80°C, storage conditions that preserved the monomeric structures, for up to 1 month. For the studies involving clearance of Aβ oligomers, Aβ1-40—reconstituted in PBS as above—was aggregated for 24 h at 37°C to generate low molecular mass oligomers, as we previously described (Fossati et al., 2010), prior to radioiodination, since labeling stably formed Aβ assemblies is known to render labeled preparations with unperturbed structural morphology preventing the generation of undesired atypical oligomers (Jungbauera et al., 2009). The resulting Aβ1-40 oligomers were immediately used for radiolabeling, for direct intracerebral injections (see below) or stored at −80°C, condition that preserved the original oligomerization structures for up to 2 weeks.

Peptide oligomerization was corroborated via electron microscopy (EM), as previously described (Fossati et al., 2010). Five microliters of either monomeric or oligomeric Aβ1-40 preparations were placed onto carbon-coated 400 mesh Cu/Rh grids (Ted Pella, Inc., Redding, CA) and stained with 1% uranyl acetate in distilled water (Polysciences, Inc., Warrington, PA). Stained grids were examined in a Philips CM-12 transmission electron microscope and photographed with a Gatan (4 × 2.7 k) digital camera at the Image Core Facility of the Skirball Institute of Biomedical Medicine, NYU School of Medicine, as described (Solito et al., 2009; Fossati et al., 2010).

### Peptide radiolabeling

Aβ1-40 in both monomeric and oligomeric forms was radiolabeled using 1,3,4,6-tetrachloro-3α-6α-diphenylglycouril pre-coated tubes (Iodogen, ThermoFisher Scientific/Pierce, Waltham, MA), following the manufacturer's specifications. All radiolabeling procedures were carried out in a Standalone Radioiodine IH-350 hood (Atlantic Nuclear, Rockland, MA). Na^125^I in NaOH (1mCi, PerkinElmer, Waltham, MA) was added to an Iodotube and combined with 20 μl of 0.1 M HCl and 60 μl of 1/10 diluted PBS. After 5-min incubation, the reaction tube was added of either monomeric or oligomeric Aβ1-40 (1 μg/μl; 100 μl), prepared as above, and the radiolabeling reaction carried out for 20 min. Following removal of free Iodine using a desalting polyacrylamide column (D-salt, cut off 1.8 kDa, Pierce), radioactivity of labeled Aβ1-40 was assessed in a scintillation counter (LS 6500 Multi-purpose Scintillation Counter, Beckman Coulter, Brea, CA). [^125^I]Aβ1-40 was analyzed by autoradiography following 16.5% SDS-polyacrylamide gel electrophoresis and subsequent direct exposure to HyBlot CL film (Denville Scientific Inc., South Plainfield, NJ). Iodinated Aβ peptides were stored at –80°C and used within 2 weeks to minimize radiolysis. Under these experimental conditions, [^125^I] incorporation rendered specific activities in the range of 3–5 μCi/μg with >98% TCA precipitable counts (Ghiso et al., 2004; Calero and Ghiso, 2005).

### Intracerebral inoculation of radio-iodinated Aβ1-40

C57BL/6 mice (Taconic Biosciences, Hudson, NY), were intracerebrally injected with Aβ1-40 preparations following institutionally approved IACUC protocols, essentially as previously reported by our laboratory (Fossati et al., 2016). Briefly, 26–30 week old mice were anesthetized by i.p. injection of a mixture of ketamine/xylazine (120 and 10 mg/kg respectively), positioned in a stereotaxic frame (David Kopf Instruments, Tujunga, CA), and intracerebrally injected (white matter of the fimbria fornix; Paxinos and Franklin Atlas coordinates: AP = −2.7, ML = −3.0 and DV = −4.0) with 1 μl of either monomeric or oligomeric [^125^I]Aβ1-40 (10 μM; flow rate: 0.2 μl/min) with the aid of a 10 μl Hamilton 701 RN syringe and a 30/2″/3S RN needle. To avoid reflux, the needle was left in position for 2 min after injection and then slowly withdrawn. At the selected time points after injection (5, 30, and 60 min), the animals were subjected to CSF collection as described below, sacrificed by trans-cardiac perfusion with PBS (2 min, medium flow pump, Fisher Scientific 13-876-2, 10 ml/min flow rate) followed by tissue harvest. Radioactivity remaining in the whole brain and cleared to CSF was assessed in an automated γ-counter (Perkin Elmer 1470).

### Intracerebral inoculation of non-radiolabeled Aβ1-40 and collection of cerebral spinal fluid

For the proteomic experiments described below, C57BL/6 mice were anesthetized and injected intracerebrally with non-labeled monomeric and oligomeric Aβ1-40 using the same methodology as described above for the inoculation of the radioiodinated peptides. At selected time points after inoculation (5, 30, and 60 min) CSF was collected from the cisterna magna following previously reported procedures (Liu and Duff, 2008) and in accordance with IACUC institutionally approved protocols. Briefly, after performing a 2 cm incision at the base of the skull, subcutaneous skin and neck muscles were separated with the aid of small animal micro-retractors (Fine Science Tools, Foster City, CA) to expose the dura mater of the cisterna magna. A siliconized borosilicate glass capillary (B100-75-10, Sutter Instruments, Novato, CA) was inserted through the dura mater, CSF was drawn into the capillary, and collected completely clear of blood. CSF samples, typically 5–8 μl per animal, were immediately frozen and stored at −80° C for future proteomic analysis.

### Immunohistochemical analysis of brain tissue post-Aβ injection

Following intracerebral injection of non-iodinated monomeric/oligomeric Aβ and CSF collection, mice were sacrificed as above by trans-cardiac perfusion with PBS (2 min) followed by 4% paraformaldehyde (5 min). Brains were harvested, post-fixed in 4% paraformaldehyde (2 h, 4°C), and cryoprotected by immersion in increasing sucrose concentrations (1 day in 15% followed by 2 days in 30% solution) before freezing as previously described (Fossati et al., 2016). Serial 8 μm-cryostat sections were collected on positively charged microscope slides and stored at −80°C until analysis. Prior to staining, sections were briefly warmed to room temperature, washed with PBS, blocked in 5% milk in PBS (1 h), and incubated in the different primary antibodies shown in Table 1 (overnight, 4°C). Slides were subsequently washed with PBS, and further incubated with the pertinent anti-rabbit, anti-mouse, or anti-goat secondary antibodies conjugated to Alexafluor 568 (red signal) or Alexafluor 488 (green fluorescence; ThermoFisher/Life Technologies, Waltham, MA; 1:200, 30 min, RT). DNA was counterstained with TO-PRO (ThermoFisher/Life Technologies; 1:2000, 10 min), and sections were mounted with aqueous mounting medium (Vectashield, Vector, Olean, NY). Images were acquired in a Zeiss LSM510 confocal microscope with a 40X/1.3 oil immersion lens and processed with Image J (https://imagej.nih.gov) and Adobe Photoshop (Adobe, San Jose, CA).

**Table 1:** **Primary antibodies for immunohistochemical studies**.

**Name**	**Species**	**Source**	**Dilution**
Aβ, 6E10	Mouse monoclonal	Covance (SIG-39320)	1:200
CD-163	Goat polyclonal	Santa Cruz (SC-18796)	1:100
E-Cadherin	Rabbit polyclonal	Abcam (15148)	1:200
Factor-VIII	Rabbit polyclonal	Abcam (6994–100)	1:200
GFAP	Rabbit polyclonal	Life Technologies (Z0334)	1:200
Iba-1	Goat polyclonal	Abcam (5076–100)	1:100
Neurotubulin	Rabbit polyclonal	Abcam (18207)	1:100

### Assessment of differential brain Aβ local degradation by immunoprecipitation and mass spectrometry

Upon intracerebral injection of non-radiolabeled monomeric/oligomeric Aβ1-40 and collection of CSF as described above, Aβ species in the biological fluid were immunoprecipitated with paramagnetic beads (Dynabeads M-280, Life Technologies) coated with 6E10 antibody (6 μg/50 μl beads), and analyzed by MALDI-ToF MS, following previously described methodologies by our laboratory (Tomidokoro et al., 2005, 2010; Hernandez-Guillamon et al., 2015). Each immunoprecipitated sample consisted of a pool of four individual CSF specimens collected from individual mice injected with non-labeled Aβ under identical conditions (*n* = 8). Samples were immunoprecipitated overnight at 4°C, beads washed with PBS containing 0.025% Tween-20 followed by 50 mM NH_4_HCO_3_, and the immunoprecipitated material subsequently eluted with 0.5 M acetic acid. After drying in a Savant SpeedVac concentrator (ThermoFisher), samples were reconstituted in 5 μl of 0.1% Formic Acid (FA) in 50% acetonitrile, and mixed with equal volumes of alpha-4-hydroxy-cinnamic-acid (AHCA; Agilent Technologies) matrix. One microliter of the final mixture was spotted in duplicate on a Bruker Daltonics MTP 384 massive target T aluminum plate and analyzed at the New York University Mass Spectrometry Core for Neuroscience using a Bruker Daltonics Autoflex MALDI-ToF mass spectrometer (Bremen, Germany) in linear mode with standard instrument settings, as described (Hernandez-Guillamon et al., 2015). External calibration was performed using human adrenocorticotropic hormone peptide 18-39 (average mass = 2465.68 Da) and insulin (average mass = 5733.49 Da). In all cases MS spectra were processed and analyzed by FlexAnalysis (Bruker Daltonics).

### Statistical analysis

ANOVA for comparison of multiple groups with Tukey *post-hoc* tests were performed using GraphPad Prism (GraphPad, La Jolla, CA). Values of *p* ≤ 0.05 were considered significant.

## Results

**Figure 1:**
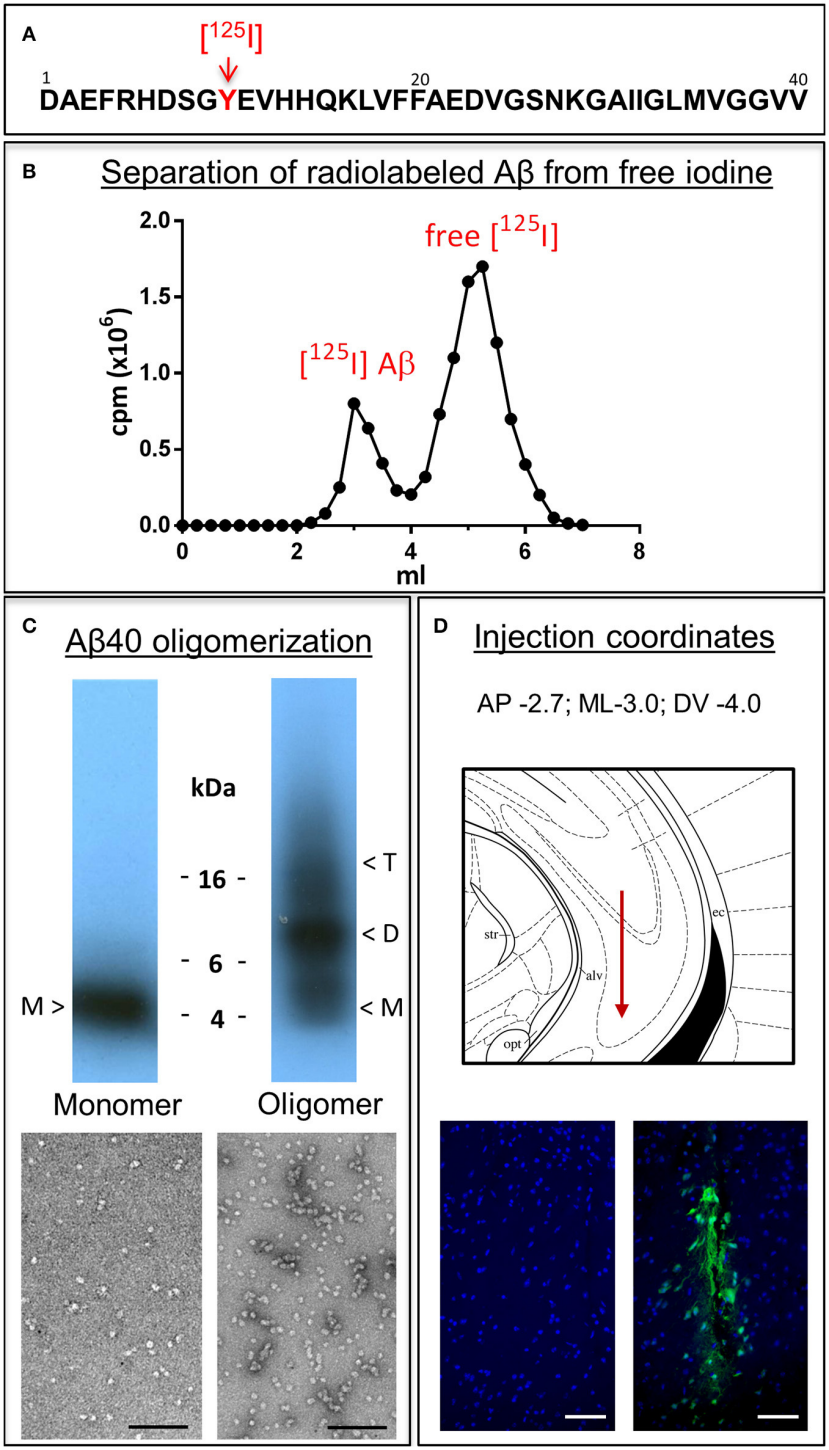
**Aβ1-40 radioiodination, assessment of peptide oligomerization and intra-cerebral injection. (A)** Amino acid sequence of Aβ1-40 highlighting the location of tyrosine 10, the target of the radioiodination procedure (red arrow). **(B)** Representative separation of [^125^I]-labeled Aβ1-40 from free iodine using a desalting 1.8 kDa cut-off polyacrylamide column. **(C)** Autoradiogram following electrophoretic separation of monomeric and oligomeric preparations of radiolabeled Aβ1-40 on 16.5% SDS-polyacrylamide gels (top) and EM images illustrating the differential conformational assemblies negatively stained with uranyl acetate (bottom). Magnification: bar represents 100 nm. **(D)** Schematic representation of the needle location for the intra-cerebral injection of Aβ1-40 preparations (top panel) and immunostaining with monoclonal anti-Aβ 6E10 followed by Alexafluor 488 conjugated secondary antibody and DAPI counterstain at the injection site demonstrating minimal—although unavoidable—tissue disruption (bottom right panel). The absence of Aβ signal in the contralateral site in animals sacrificed immediately after Aβ injection corroborates the specificity of the immunostaining (bottom left panel). Magnification: bar represents 100 μm.

Monomeric and oligomeric of Aβ1-40 preparations were labeled with Na[^125^I] at the tyrosine residue located at position 10 of Aβ (Figure 1A), as described above in Material and Methods. The use of a 1.8 kDa cut off desalting column allowed the removal of free iodine from radio-iodinated Aβ, as illustrated in Figure 1B by the clear separation between peaks in a representative experiment. Autoradiography after SDS-gel electrophoresis confirmed the respective monomeric or oligomeric composition of each respective preparation. As indicated in the pertinent autoradiograms shown in Figure 1C, monomeric preparations displayed a single 4 kDa band whereas oligomeric preparations consisted of low molecular mass Aβ species, exhibiting monomeric, dimeric, trimeric, and tetrameric components with predominance of dimeric forms. The monomeric or oligomeric nature of the preparations was corroborated via EM in parallel experiments performed using non-labeled counterparts. As illustrated in Figure 1C, monomeric preparations rendered scattered globular structures 4–6 nm in diameter whereas oligomeric preparations contained a higher number of the same globular structures in many cases associated in small groups containing 2–4 globular components. No evidence of protofibrillar formation was noticed at this time-point, in agreement with our previous reports (Solito et al., 2009; Viana et al., 2009; Fossati et al., 2010). Classic protofibrils (short rods of < 150 nm in length and 4–6 nm in diameter) did not appear before an additional 24 h incubation in our experimental conditions, providing the necessary safety net of structural stability for the 1 h window of our intra-cerebral injection experiments. Both monomeric and oligomeric preparations were intra-cerebrally inoculated into C57BL/6 mice to evaluate their respective efflux from brain. The schematic representation in Figure 1D depicts the injection coordinates and the needle position whereas immunostaining at the actual injection site using anti-Aβ monoclonal 6E10 and Alexafluor 488 conjugates demonstrates minimal—although unavoidable—tissue disruption (right panel). Specificity of the immunostaining is indicated by the absence of Aβ signal in the contralateral site in animals sacrificed immediately after inoculation (left panel).

**Figure 2:**
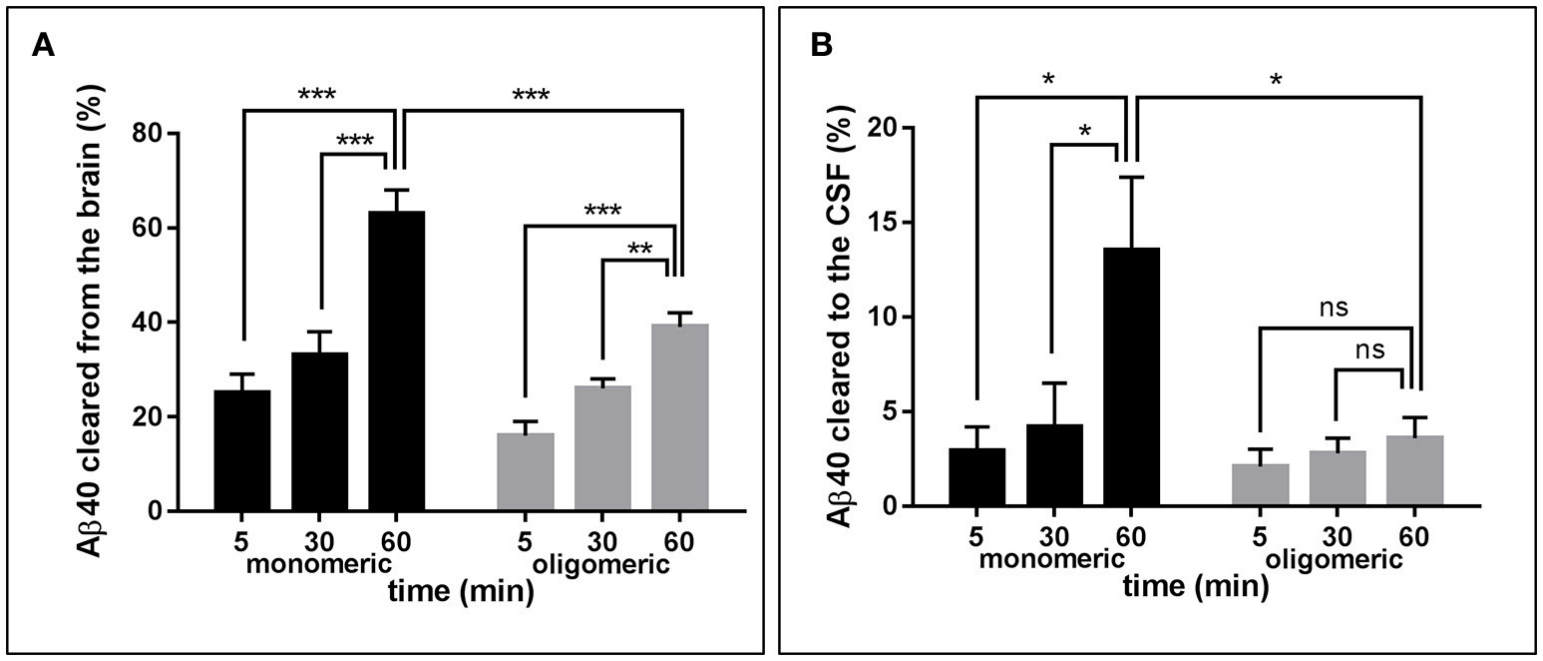
**Brain clearance of radiolabeled Aβ species**. **(A)** Clearance of monomeric (black bars) and oligomeric (gray bars) [^125^I]Aβ1-40 species removed from the brain at three different time-points. **(B)** Monomeric (black bars) and oligomeric (gray bars) [^125^I]Aβ1-40 species cleared to the CSF evaluated at three different time-points. In all cases bars illustrate percentage cleared relative to total injected radioactivity; values represent mean ± *SD* obtained from inoculation of 5–7 mice (ANOVA and Tukey *post-hoc* test; ns, not significant; ^*^p < 0.05; ^**^p < 0.01; ^***^p < 0.001).

Figure 2A depicts the time-related clearance of monomeric and LMW oligomeric [^125^I]Aβ1-40 from brain interstitial fluid, estimated based on the radioactivity remaining in the whole brain at 5, 30, and 60 min post-injection compared to the total injected radioactivity. Brain efflux of monomeric Aβ1-40 was fast, with ~25% of the peptide cleared in only 5 min and >60% cleared within 60 min. Retention of LMW oligomers was consistently higher, with only ~15% of the injected material eliminated in the first 5 min and < 40% at 60 min. Comparison of brain clearance levels for monomeric vs. oligomeric forms of [^125^I]Aβ1-40 evaluated 60 min after intra-cerebral injection clearly demonstrated that peptide oligomerization significantly increased brain retention (*p* < 0.001). Clearance from interstitial fluid to the CSF was evaluated by quantitating radioactivity in the CSF 5, 30, and 60 min post-Aβ intracerebral injection (Figure 2B). CSF was collected through a cisterna magna puncturing technique without blood contamination, as corroborated by dot blot analysis probed with antibodies immunoreacting with apolipoprotein-B and α2-macrogloblin, blood proteins not present in the CSF (data not shown). Only a fraction of the material removed from brain reached the CSF in a time-dependent manner. In the case of monomeric Aβ1-40, the levels cleared to the CSF increased from ~3% at 5 min to an average of 13% at 60 min. In contrast, LMW oligomeric Aβ1-40 was cleared less efficiently, with only ~2% present in the CSF after 5 min, and ~4% at 60 min. Comparative evaluation of [^125^I]-Aβ cleared to the CSF at 60 min indicates a statistically significant decrease of about 3-fold in oligomeric Aβ removal compared to the values obtained for the monomeric form of the peptide (*p* < 0.05) and in line with the results illustrated in Figure 2A for overall brain efflux.

**Figure 3:**
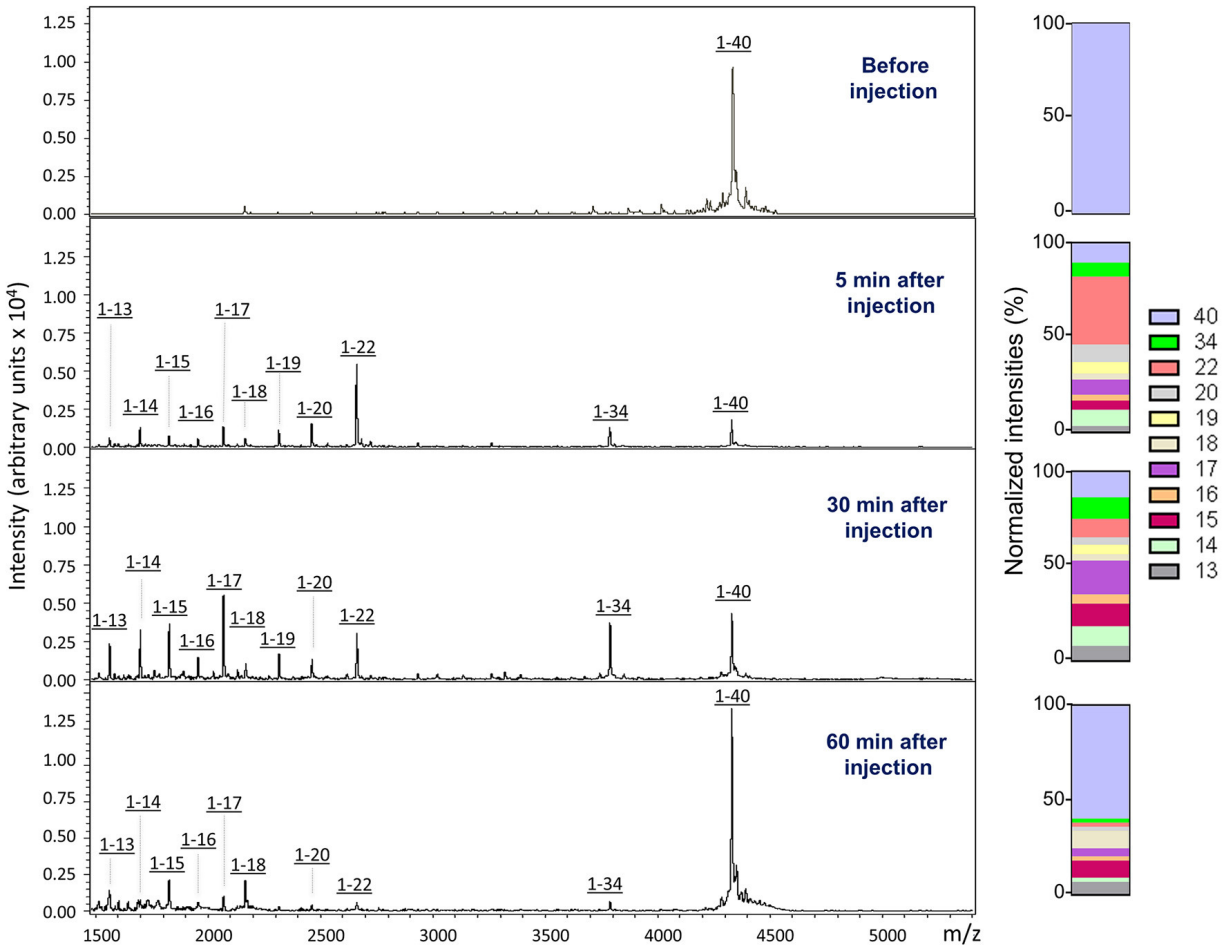
**Aβ species cleared to the CSF assessed by a combination of immunoprecipitation and mass spectrometry**. MALDI-ToF spectra and normalized ion counts of the monomeric Aβ1-40 used for the experiments prior injection (top). MS profile of the material immunoprecipitated from CSF at different time points (5, 30, and 60 min) post injection identify multiple C-terminally degraded proteolytic fragments. Immunoprecititation was performed in pooled CSF samples from 4 mice injected under the same experimental conditions in 2 independent experiments (*n* = 8) and spotted in duplicate in the mass spectrometer aluminum plates.

In order to biochemically analyze the Aβ species cleared to the CSF, a targeted mass spectrometric analysis of Aβ was conducted on CSF collected from a parallel set of mice 5, 30, and 60 min after intra-cerebral injection of non-radiolabeled monomeric and oligomeric forms of Aβ1-40. Immunoprecipitation of CSF samples with paramagnetic beads coated with monoclonal anti-Aβ 6E10 antibodies followed by MALDI-ToF mass spectrometry analysis revealed the presence of heterogeneous C-terminally truncated Aβ species in addition to full-length Aβ1-40, suggestive of a fast enzymatic degradation by brain resident enzymes. Figure 3 depicts the differential pattern exhibited by the intact injected peptide—a single peak with an experimental mass of 4329.1Da (top panel)—in comparison with the multiple degradation fragments retrieved in CSF at the different time-points (lower panels). The relative intensity ratios between the intact peptide and each of the major proteolytically- generated fragments assessed at the different time points indicate that the CSF truncated Aβ species became comparatively less abundant with time while the peak pertaining to the intact peptide, a relatively minor component at 5 min, increased as the time after injection progressed and was the predominant form at 60 min (Figure 3). These differences in the CSF Aβ heterogeneity—highly dependent on the time of sample collection following the intra-cerebral Aβ injection—strongly suggest that C-terminally degraded Aβ fragments are cleared into and out of the CSF more efficiently than the full length peptide—likely reflecting their higher solubility and lower tendency to aggregate (Cabrera et al., unpublished)—and supporting the beneficial role for local catabolism in brain Aβ efflux. Whether a decreased ratio of Aβ-truncated fragments at longer time points after injection also reflects a preferential involvement of other Aβ-removal mechanisms including perivascular drainage, glymphatic pathway or meningeal lymphatic vessels, and/or simply results from the focal exhaustion of available local enzymes remain to be elucidated.

**Figure 4:**
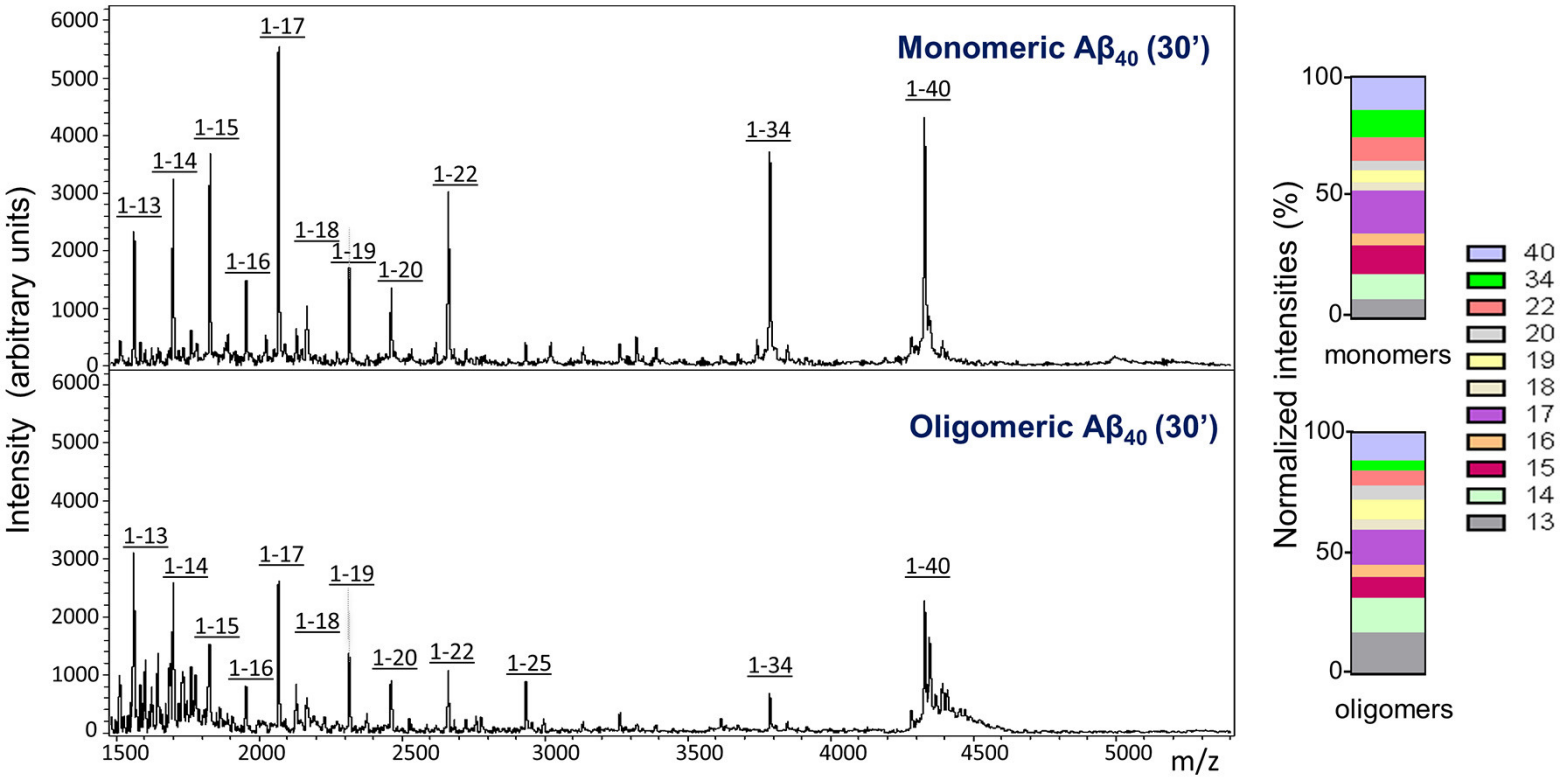
**Comparative analysis of Aβ species cleared to the CSF after intracerebral injection of monomeric and oligomeric Aβ1-40**. MALDI-ToF spectra and normalized ion counts on CSF collected 30 min after intracerebral injection of comparable amounts of monomeric (top panel) and oligomeric (bottom panel) Aβ1-40 immunoprecipitated with anti-Aβ monoclonal antibody 6E10. For the immunoprecipitation, CSF was pooled from 4 mice injected under the same experimental conditions. Data are representative of 2 independent experiments (*n* = 8) spotted in duplicate in the mass spectrometer aluminum plates.

Similar to what was observed for the injected monomeric Aβ1-40, analysis of CSF collected from mice injected with LMW oligomeric forms of the peptide displayed, in addition to the full length peptide, a heterogeneous collection of C-terminally truncated species (Figure 4). It should be noted that under the experimental conditions of the mass spectrometry assay, which render non-covalent binding unobservable (Woods et al., 1995), the amyloid molecules are detected at the molecular mass corresponding to the monomeric species, independent of their aggregation state, as we previously reported (Tomidokoro et al., 2010); therefore, the full-length peak is detected with the same experimental mass regardless the use of monomers or LMW oligomers in the experiments. Visual evaluation of the CSF Aβ species resulting from the inoculation of monomeric vs. oligomeric Aβ1-40 at 30 min post-injection (Figure 4, top and bottom panels, respectively) revealed a similar heterogeneity—full-length peptide coexisting with the same C-terminally degraded Aβ fragments—indicative that, irrespective of the aggregation status of the injected Aβ, the same peptide bonds were targeted for enzymatic degradation. Analysis of the relative intensity ratios corresponding to the intact peptide and to each of the major proteolytic fragments generated from the monomeric and the LMW oligomeric species reinforced the similarity of the proteolytic process (Figure 4, normalized intensities). While absolute signal intensity between samples in MALDI-ToF is not reliably quantitative, lower signal intensity for the cleared oligomeric injected forms are consistent with an indication that Aβ1-40 full-length oligomers are not removed as efficiently to the CSF as the monomeric form, in agreement with results illustrated in Figure 2. The lower susceptibility of oligomeric injected forms of Aβ to enzymatic degradation suggested by the lower signal intensity of the C-terminally degraded derivatives—consistent with previously reported studies for most Aβ-degrading enzymes (Morelli et al., 2003; Haass and Selkoe, 2007; De Strooper, 2010)—may also account, at least in part, for the lower clearance rate observed in our experimental paradigm for oligomeric Aβ species shown in Figure 2.

**Figure 5:**
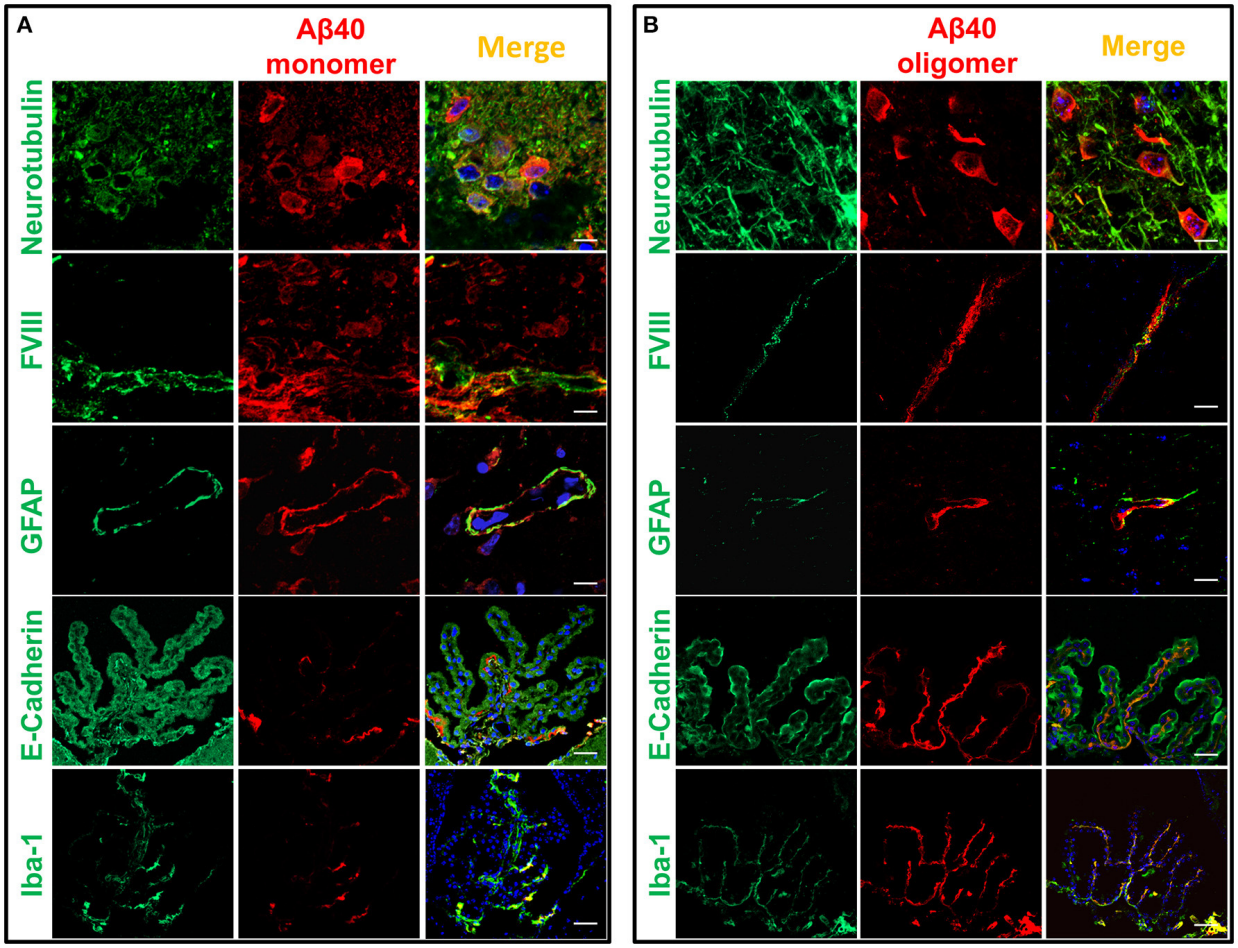
**Cellular localization of monomeric and oligomeric Aβ after intracerebral injection**. Immunofluorescence microscopy analysis of serial frozen sections from brains of mice injected with monomeric **(A)** and oligomeric **(B)** Aβ illustrates the co-localization of Aβ (red signal) with cell specific markers of neurons (neurotubulin), endothelial cells (factor VIII), astrocytes (GFAP), choroid plexus epithelium (E-cadherin), and activated microglia (Iba-1), all in green fluorescence. Co-localization is highlighted by the yellow fluorescence in the merged images. Magnification: bar represents 10 μm in the neurotubulin stainings, 20 μm for the GFAP stainings, and 30 μm in the case of the FVIII, E-cadherin and Iba-1 stainings.

Immunofluorescence confocal microscopy studies in brain tissues harvested 60 min following intracerebral injection of non-labeled Aβ1-40 provided insight into the cellular mechanisms involved in Aβ uptake. Brain tissue sections were co-immunolabeled with antibodies recognizing specific cell types in conjunction with monoclonal anti-Aβ antibody 6E10 and co-localization of the respective signals evaluated at the site of the injection. The images described below were captured from the motor cortex adjacent to the injection site. Although the distance from the injection point varied among the different stainings, the images shown in Figure 5 were typically acquired within a 400 μm range from the injection site. Figure 5A illustrates the localization of injected Aβ monomers to neuronal cells highlighted by neurotubulin staining consistent with recent reports demonstrating the capability of neurons to uptake ISF Aβ through receptor-mediated endocytosis by the low-density lipoprotein receptor-related protein 1 (LRP1; Kanekiyo et al., 2013). Aβ was also present in the cerebral vasculature, as indicated by its co-localization with endothelial cells decorated by their reactivity with Factor VIII, suggestive of Aβ clearance across the BBB, a well study pathway for the removal of Aβ monomers (Shibata et al., 2000; Bell et al., 2007; Deane et al., 2009), or along the peri-/para-vascular tracks, as recently reported (Morris et al., 2016). The immunohistochemical approach also illustrates the co-localization of Aβ with astrocytes surrounding the vasculature—as indicated by the overlapping staining pattern of Aβ and the astrocytic marker GFAP—indicative of the recently described glymphatic pathway involving the aquaporin 4-mediated ISF bulk flow, as one of the mechanisms involved in the clearance of the injected Aβ. Analysis of the choroid plexus epithelium with antibodies recognizing E-cadherin indicates the presence of the intra-cerebrally injected Aβ also at this barrier structure and in accordance to the presence of degraded and intact Aβ in the CSF illustrated in Figures 2–4. Notably, Aβ reactivity was also associated with activated microglia within the choroid plexus, as highlighted by the overlapping signals of Aβ with the Iba-1 microglial marker. The immunoreactivity of 6E10 does not distinguish between monomeric and oligomeric forms of Aβ; thus, we used this antibody in immunohistochemical studies to also assess the localization of the injected oligomeric preparations. As indicated above, the Aβ1–40 oligomers used in the experiments remained stable during the 1 h window of the intra-cerebral injection procedure requiring an additional 24 h incubation to render protofibrillar structures (Fossati et al., 2010). Figure 5B illustrates the comparable overlapping of signals recognizing the injected LMW oligomeric Aβ with neuronal, endothelial, and astrocytic cells as well as within choroid plexus structures and associated activated microglia, suggesting the involvement of comparable routes for the efflux of monomeric and LMW oligomeric Aβ counterparts.

## Discussion

Over the last decade increasing evidence indicates that one of the main mechanisms leading to brain Aβ accumulation and likely contributing to AD is a defective clearance of the protein from the brain, which affects the delicate balance between degree of Aβ production, dynamics of aggregation, and rate of brain efflux. Glial phagocytosis, local enzymatic degradation, efflux through the BBB, transport to the cerebrospinal fluid, peri- and para-vascular drainage along with the more recently described glymphatic system and meningeal lymphatic vessels are among the mechanisms under current investigation and with demonstrated participation in brain Aβ removal (Deane et al., 2004, 2008, 2009; Iliff et al., 2012; Morris et al., 2014, 2016; Bakker et al., 2016). Of all these pathways, clearance across the BBB is definitely the most studied and one of the most significant contributors to Aβ brain removal accounting for ~75% of the overall Aβ efflux in humans (Roberts et al., 2014). Once cleared to the blood stream and consistent to its very low and steady concentration in plasma, full-length Aβ has a very short half-life: ~3 min in mice, similar to that of insulin or oxytocin (Ghiso et al., 2004). Systemic Aβ catabolism occurs mainly in the liver through hepatocyte uptake and proteolytic degradation followed by secretion of the intact peptide and its proteolytically-derived fragments into the bile (Ghiso et al., 2001). Consistent with its short half-life in the periphery, the data presented herein unmistakably demonstrate that Aβ is also cleared from the brain at a fast rate and in a time-dependent manner. About 25% of the monomeric peptide is cleared from the brain only 5 min after the intra-cerebral injection whereas the amount of Aβ eliminated increases steadily with time reaching levels of >60% at 60 min, the longest time-point evaluated in these studies. Notably, transport across the brain-CSF barrier plays a significant part in Aβ elimination as ~3% of intra-cerebrally injected ^125^I-Aβ was found in CSF after 5 min, and 13% 1 h post-injection, consistent with previous reports—also employing fresh monomeric preparations of radiolabeled Aβ1-40—which estimated Aβ clearance to the CSF in 10–15% (Shibata et al., 2000; Deane et al., 2003).

An impressive amount of research supports a pathogenic role for Aβ oligomerization in AD. Small intermediate soluble Aβ oligomers, similar to those employed in the current studies and primarily consisting of SDS-resistant dimers and trimers (Haass and Selkoe, 2007), have been implicated in synaptic dysfunction and neuronal loss leading to the progressive dementia associated in later stages of the disease with extensive Aβ pathology (Haass and Selkoe, 2007; Walsh and Selkoe, 2007; Li et al., 2009; Jin et al., 2011; Shankar et al., 2011). It is currently hypothesized that accumulation of these Aβ oligomeric assemblies during the disease progression translates in increasingly severe and permanent changes in synaptic function. The ^125^I-Aβ1-40 clearance studies, showing higher brain retention and an ~ 3 -times lower efflux to the CSF for oligomeric Aβ in comparison to the values observed for the monomeric form, add another layer of pathogenic significance to these assemblies. Through a delayed brain removal, oligomeric species—once formed—have higher capability to exert their pathogenic activity. Furthermore, since the process of multimerization is concentration dependent, the persistence of oligomeric forms of Aβ within the brain is likely to exacerbate the assembly of higher molecular mass species and/or recruit soluble forms of Aβ into multimolecular species in a nucleation/seeding effect which is increasingly recognized as a significant contributor to the pathogenesis of neurodegenerative disorders (Kane et al., 2000; Meyer-Luehmann et al., 2006).

Targeted mass spectrometric analysis of the CSF collected at different time points after the post-intracerebral Aβ injections consistently demonstrated that Aβ is cleared to the CSF not only as a full-length peptide but also in the form of numerous C-terminally truncated fragments, indicative of catabolic cleavage by Aβ-degrading brain resident enzymes. This enzymatic degradation takes place very fast, as evidenced by the presence of the Aβ degradation products after only 5 min post-injection. Comparative analysis of the Aβ catabolic footprints with the time of CSF collection elapsed after the intracerebral injection provides a clear indication of the preferential early removal of these C-terminally degraded fragments in comparison to the full-length Aβ1-40 providing support for an important role of enzymatic degradation in the fast and efficient brain Aβ clearance. Along this line, the higher retention exhibited by Aβ oligomeric species may well relate not only to a decreased efficiency in the efflux of these species *per-se* but also to a lower susceptibility of these assemblies of being cleaved by brain resident enzymes, as evidenced in the current studies by the lower ratio of cleaved Aβ forms present in the CSF samples of animals intracerebrally injected with Aβ1-40 oligomers. Indeed, the lower susceptibility of Aβ aggregated species to enzymatic cleavage has been previously documented—mostly in *in vitro* paradigms—not only for Aβ, but also for other amyloid molecules associated with different cerebral amyloidoses like the chromosome XIII dementias, as well as for systemic forms of the disease (Haass and Selkoe, 2007; Rostagno et al., 2007; De Strooper, 2010).

**Figure 6:**
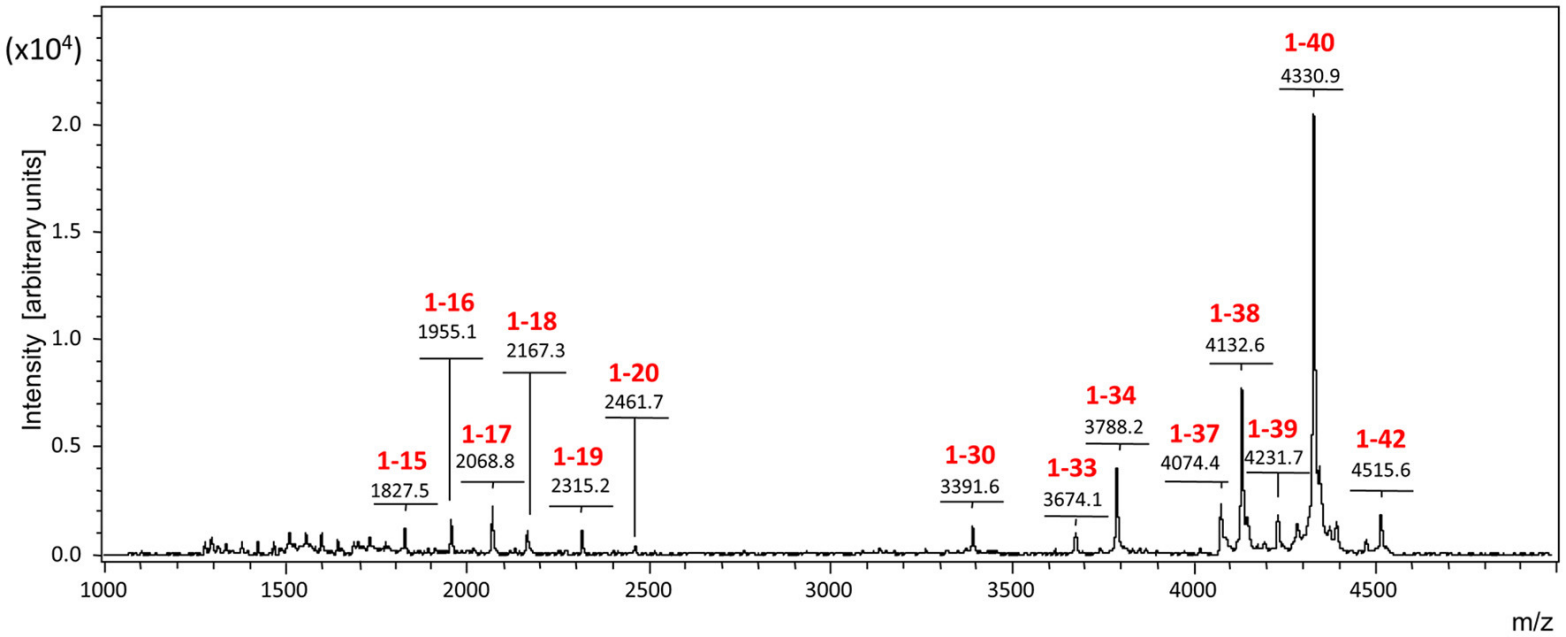
**The Aβ-peptidome in normal human CSF**. The spectrum highlights the C-terminal heterogeneity of the Aβ species present in the CSF, extending far beyond the classic Aβ1-42/Aβ1-40 dichotomy and reflecting the proteolytic action of local resident enzymes.

Enzyme-mediated clearance has received considerable attention during the last decade, and many of the multiple enzymes constituting the proteolytic machinery of the brain have shown potential to participate in Aβ catabolism, among them neprylisin, insulin degrading- and endothelin converting-enzymes, plasmin as well as matrix metalloproteases (Selkoe, 2001; Morelli et al., 2002; Wang et al., 2006; Miners et al., 2008; Saido and Leissring, 2012; Hernandez-Guillamon et al., 2015). Reduced levels and/or decreased catalytic activity of these Aβ-degrading enzymes as a result of age, genetic factors, and specific disease conditions have been proposed to affect Aβ accumulation, an issue well-documented in murine models in which gene deletion of different proteases translate into increased levels of Aβ deposition (Selkoe, 2001; Vardy et al., 2005; Wang et al., 2006). Evidence of a genetic association of these proteases with AD has only been reported for a few enzymes albeit no consensus exists to the moment with regard to the importance or reproducibility of these associations in the general AD population (De Strooper, 2010). Nevertheless, in spite of this elusive information, the relevance of these enzymatic processes for brain clearance is supported in part by the current studies which demonstrate the preferential elimination of truncated C-terminal species to the CSF as well as by previous reports highlighting the higher solubility and non-toxic characteristics of the resulting truncated species (Hernandez-Guillamon et al., 2015), features that point out to a beneficial role of Aβ enzymatic processing for AD pathogenesis. The heterogeneous CSF Aβ profile generated by enzymatic catabolism of injected Aβ1-40 correlates well with the heterogeneity of Aβ species present in brain tissue and CSF biological samples. As illustrated in Figure 6 for human CSF in normal individuals, Aβ heterogeneity extends far beyond the classic Aβ1-40/Aβ1-42 dichotomy and encompasses numerous Aβ truncated forms, primarily cleaved at the C-terminus in CSF, and both at the C- and N-terminal ends in brain specimens (Portelius et al., 2006; Tomidokoro et al., 2010).

Immunofluorescence confocal studies performed following intrahippocampal Aβ inoculation demonstrated localization of the peptide with neurons, at microvessels in association with endothelial and astrocytic components, as well as at the chroid plexus epithelium. This peptide localization, which was also visualized in the case of the inoculation of Aβ oligomers, highlights different routes of cellular uptake, and clearance. Internalization of Aβ by neurons can be clearly observed in the experiments described herein, highlighting an important route of cellular uptake, consistent with recent reports describing the capability of neurons to uptake Aβ from interstitial fluid (Kanekiyo et al., 2013). Indeed, although cellular uptake by astrocytes, microglia, and vascular smooth muscle cells have been shown to participate in Aβ elimination (Wyss-Coray et al., 2001, 2003; Koistinaho et al., 2004; Kanekiyo et al., 2012) neuronal uptake has received much less attention in spite of the high vulnerability of these cells to Aβ-mediated toxicity. Recent data indicate that neuronal LRP-1—a known endocytic receptor for multiple ligands, including Aβ (Kanekiyo et al., 2013) and predominantly expressed in the postsynaptic region and the cell body (Bu et al., 1994; May et al., 2004)—mediates neuronal Aβ uptake and subsequent degradation primarily by lysosome-dependent pathways (Kanekiyo et al., 2013).

Immunofluorescence studies post-injection also demonstrated the presence of Aβ in the cerebral microvasculature, as indicated by colocalization of the peptide with endothelial cells highlighted by their reactivity with Factor VIII, suggestive of Aβ clearance across the BBB, a well-studied pathway for the removal of Aβ monomers (Shibata et al., 2000; Deane et al., 2009). The BBB, responsible for the overall brain and CNS maintenance, is a highly specialized structure which maintains neuronal homeostasis by regulating the flux of electrolytes, metabolites, toxic molecules, and circulating immune cells between the blood stream and the brain parenchyma (Abbott et al., 2010; Di Marco et al., 2015). It is primarily formed by basement membranes and capillary endothelial cells which adhere to one another through tight and adherens junctions, albeit astrocytes, and pericytes—topographically located in close contact with the endothelium (Allt and Lawrenson, 2001; Armulik et al., 2005)—are essential contributors to the BBB phenotype. Through this permeability barrier, the brain efficiently protects itself from harmful compounds and precisely regulates its microenvironment (Cecchelli et al., 2007). Small lipid-soluble molecules, such as oxygen and carbon-dioxide can diffuse freely across the BBB, whereas the exchange of larger molecules occurs by either active transport through the cell body (transcytosis) or by paracellular transport (Zlokovic, 2011; Lyros et al., 2014; Di Marco et al., 2015). As macromolecules, Aβ peptides cross the BBB by active transport. The influx from the blood to the brain is mediated by the luminal residing receptor for advanced glycation end-products (RAGE; Deane et al., 2003) whereas when complexed to its carrier protein clusterin (apolipoprotein J) within the high density lipoproteins, Aβ is transported into the brain through the clusterin receptor megalin (gp330 or LRP-2; Zlokovic et al., 1996). LRP-1, predominantly localized on the abluminal side of the cerebral endothelium (Shibata et al., 2000) as well as P-glycoprotein (P-gp)—an ATP-dependent efflux pump with broad specificity also abundant in the brain capillary endothelium—are the main receptors responsible for Aβ efflux from brain-to-blood (Shibata et al., 2000; Deane et al., 2003; Candela et al., 2010; Di Marco et al., 2015). More recently, a novel protein in brain endothelial monolayers has also been shown to play a role in the brain clearance of Aβ. This protein, known as PICALM (phosphatidylinositol binding clathrin assembly protein), is abundantly expressed in brain capillary endothelium (Baig et al., 2010; Parikh et al., 2014), and has been linked to AD in recent genome wide association studies (Harold et al., 2009; Tanzi, 2012; Lambert et al., 2013). Through the PICALM/clathrin-dependent internalization of Aβ bound to LRP-1, this newly reported pathway also contributes to Aβ endothelial transcytosis and brain clearance.

Endothelial cells lining the cerebral microvasculature are the primary cellular element forming the BBB albeit pericytes and astrocytes are essential contributors to the physiological function of this barrier (Zlokovic et al., 1996; Deane et al., 2008). In particular astrocytes, which are in close anatomical proximity to the brain microvasculature as their endfeet form a lacework surrounding the outer surface of the endothelium (Abbott, 2002), are crucial for the development of the specialized BBB phenotype. This cross-talk with the brain endothelium is now known to involve complex cell–cell exchange of chemical signals responsible for the induction of the specific features and the modulation of the cellular physiology of the BBB. The immunohistochemical approach in the current work demonstrates colocalization of Aβ with astrocytes surrounding the microvasculature, as indicated by the overlapping staining pattern of Aβ and the astrocytic marker GFAP suggestive of a participation of these cells in Aβ clearance at the BBB. Indeed, astrocytes have been shown as capable of binding and internalizing Aβ in *in vitro* systems using live-cell imaging methodologies although the precise mechanisms involved—notably significantly less efficient for the clearing of Aβ fibrillar assemblies—remain to be elucidated (Nielsen et al., 2010).

Analysis of the choroid plexus epithelium by immunofluorescence studies indicates the presence of the intracerebrally injected Aβ at this barrier structure. Notably, Aβ reactivity was also associated with activated microglia within the choroid plexus as highlighted by the overlapping signals of Aβ with the Iba-1 microglial marker in association with this epithelial barrier. The participation of microglia, the brain's tissue macrophages and the primary immune effectors within the CNS, in the clearance of Aβ has been previously reported (Mandrekar et al., 2009; Nielsen et al., 2010). These highly dynamic cells, responsible for normal tissue maintenance, continually police the brain extracellular environment taking up soluble nutrients (Nimmerjahn et al., 2005). Microglia uptake Aβ peptides through macropynocytic mechanisms, rapidly trafficking the peptides into late endolysosomal compartments for subsequent degradation, actively participating in the maintenance of Aβ homeostasis (Mandrekar et al., 2009). The present data provide support for the relevance of microglia-mediated uptake at the level of the choroid plexus structures. The bulk flow of ISF into the CSF through these specialized epithelium—which notably shares many of the receptors involved in BBB clearance, including LRP-1 and P-gp (Dietrich et al., 2008; Behl et al., 2009)—has been reported to contribute 10–15% of brain Aβ clearance, in agreement with the current report using radiolabeled Aβ1-40 and supported by the localization of Aβ to the epithelial choroid barrier.

Notably, the role of CSF in clearing Aβ along with waste and toxic elements from the brain have become increasingly more complex in the last few years with new evidence highlighting the relevance of peri- and para-vascular intracerebral transport routes (Bakker et al., 2016), the description of the glymphatic system (Iliff et al., 2012), and the discovery of meningeal lymphatic vessels (Aspelund et al., 2015; Louveau et al., 2015), the latter a potential clearance route yet to be studied. The ISF bulk-flow clearance removes ISF—which contains Aβ as well as other solutes and waste products—from the interstitium into the CSF and perivascular space aided by the force resulting from vessel pulsations. Evidence of drainage of ISF along perivascular pathways, driving proteins, and fluid along blood vessel walls were demonstrated early on through studies focusing on the elimination of tracers injected into the brain parenchyma (Bakker et al., 2016). These tracers diffuse through extracellular spaces of the brain along basement membranes of capillaries to drain out of the brain along basement membranes in the tunica media of arteries and into the cervical lymph nodes (Weller et al., 2008). Adding to the complexity of ISF/CSF intracerebral circulation, a recycle of CSF through the brain ISF has recently been described (Iliff et al., 2012). This pathway, known as glymphatic transport system, is dependent on the expression of the water channel protein aquaporin 4 by astrocytes and on efficient arterial pulsations. Intra-cisternally injected Aβ has been shown to be transported along this route, a mechanism significantly reduced in aquaporin 4 knock-out mice. Although the relative contributions of each of these systems to overall clearance remains to be elucidated, these mechanisms synergistically contribute to the elimination of extracellular Aβ from the brain and alterations in any of these closely interlinked mechanisms are likely to contribute to Aβ accumulation. The intricate circulation of CSF is likely to influence the clearance of other pathogenic protein in AD, the intracellular neuronal protein Tau, which although not the focus of the current studies has demonstrated relevance for the disease pathogenesis. Indeed, Tau clearance is significantly less well-studied than Aβ clearance, but it also seems to be less complex. Transporters specifically mediating the passage of Tau through the BBB have not been identified, which seem to suggest that the protein does not undergo clearance through the BBB under normal physiological conditions. Instead, tau is thought to be cleared from the brain primarily by degradation, by ISF bulk flow into the CSF, and by CSF absorption (Chesser et al., 2013; Iliff et al., 2014; Ueno et al., 2016). As it is the case of Aβ and brain metabolic/waste products, CSF absorption into the blood at the arachnoid villi as well its reentry into the ISF by paravascular mechanisms and aquaporin mediated circulation within the glymphatic system seem to contribute to Tau clearance (Iliff et al., 2014; Ueno et al., 2016).

It is interesting to mention that most—if not all—of the physiological mechanisms currently thought to participate in brain clearance are severely affected by aging, feature that may correlate with the age-associated characteristics of AD. In this sense, the stiffening of cerebral arteries with age, lead to reduced amplitude of pulsations and to a decreased efficacy in perivascular and ISF drainage pathways, deficiencies accentuated when vessels are compromised by amyloid deposits in cerebral amyloid angiopathy which further impede the drainage of soluble Aβ (Weller et al., 2008; Hawkes et al., 2011). A significant decrease in CSF production and sluggish flow is also observed in aging. This dwindling CSF dynamics, which greatly harms the interstitial environment of neurons, is aggravated in AD in which the expanding CSF space reduces CSF turnover rate further compromising the sink action to clear harmful metabolites, among them Aβ (Johanson et al., 2008; Zeng et al., 2012), an effect also impacted by the age-associated decreased functionality of the choroid plexus-CSF system (Lustbader et al., 2004; Kheterpal et al., 2006; Behl et al., 2009), as well as by the age-related decrease in the levels of the Aβ efflux transporters both at the choroid plexus and the BBB. All these changes individually or concurrently have undisputed relevance for brain clearance and a complete understanding of these mechanisms may provide new insights leading to the delay or even the prevention of the onset of AD.

Overall, the data presented herein, through intracerebral injection of well-defined monomeric and low molecular mass oligomeric Aβ assemblies in CB57BL6 mice, highlight the fast physiologic brain elimination of the peptide providing a clear indication of the detrimental role of oligomerization for brain Aβ efflux. The findings clearly demonstrate that physiologic clearance of brain interstitial Aβ follows local and systemic paths and that in addition to the blood-brain barrier, local enzymatic degradation, and the bulk flow transport through the choroid plexus into the CSF play a crucial role. The complexity of the interlinked Aβ brain removal mechanisms, encompassing different cell populations and in some cases overlapping cellular pathways explain to a certain point the lack of success of therapeutic strategies targeting individual components of this multiplex cascade. Our studies highlighting the diverse factors influencing brain clearance and the various routes of elimination open up new research opportunities for the understanding of altered mechanisms triggering AD pathology and for the potential design of combined therapeutic strategies.

## Author contributions

JG designed research; FM and PG performed experimental work; AR and JG analyzed data, interpreted results, and wrote the paper; SB performed MS analysis; GS provided expertise in radioactivity assessment and quality control; TN analyzed MS studies; FM, PG, and TN edited the manuscript.

### Conflict of interest statement

The authors declare that the research was conducted in the absence of any commercial or financial relationships that could be construed as a potential conflict of interest.
